# A dataset of riverine nitrogen yield across watersheds in the Conterminous United States

**DOI:** 10.1038/s41597-024-03552-1

**Published:** 2024-06-29

**Authors:** Yiming Wang, Xuesong Zhang, Kaiguang Zhao

**Affiliations:** 1 Oak Ridge Institute for Science and Education, Oak Ridge, TN 37830 USA; 2 Ohio Agricultural Research and Development Center, School of Environment and Natural Resources, The Ohio State University, Wooster, OH 44691 USA

**Keywords:** Element cycles, Pollution remediation

## Abstract

Riverine nitrogen is a pivotal determinant influencing water quality in inland and coastal waters. Despite the recognized utility, no spatially-explicit data on riverine nitrogen yield is available for large parts of the world, thus hindering our ability to identify the contributors to riverine nitrogen and understand aquatic nitrogen cycling. To fill the data gap for the United States, here we (1) compiled 294,996 total nitrogen (TN), 225,827 nitrate (NO_3_^−^), 204,015 ammonium (NH_4_^+^), and 158,837 total organic nitrogen (TON) concentrations, with concurrent streamflow data, across the Conterminous United States (CONUS), (2) estimated riverine nitrogen loads for over 1,800 hydrological stations, (3) derived the spatial distribution of annual riverine nitrogen yield by leveraging river and catchment connectivity information contained in the National Hydrography Dataset plus (NHDPlus), and (4) characterized nonpoint-source TN loads by excluding point-source loads. This new spatial dataset quantifies spatial sources of nitrogen yield from point and non-point sources (e.g., up to 36% from point sources across the U.S.) and serves as ground-truthing to validate water quality models.

## Background & Summary

Nitrogen (N) from point or nonpoint sources has been emerging as a predominant contributor causing suboptimal water quality in eutrophic streams and rivers in the United States^1,2^ and across the globe^3^. Escalations in riverine nitrogen concentrations have become pervasive across myriads of watersheds, attributed largely to shifts in land utilization practices, modifications in agricultural methodologies, and climate-induced variations^4,5^. Globally, about 118 tera-grams of nitrogen (TgN) per year was applied as fertilizers^6^, and 18% of them was leached into oceans via river runoff^7^. Recent estimates of riverine nitrogen loss into oceans showed a pronounced increase from 17 TgN per year during the preindustrial era to 37.6 TgN per year in the last decade^8^, as particularly evident for major rivers such as the Amazon, Mississippi, and Yangtze^9^. Elevated nitrogen in rivers was documented to transform and impair many riverine ecosystems and estuaries across the world^10,11^, urging for concerted efforts to manage nutrients and mitigate eutrophication as well as for more publicly accessible data to support such efforts.

Extensive data on nitrogen concentrations and streamflow exist for many parts of the world. But existing data syntheses or analyses focused mostly on large-scale accounting (e.g., N loss into the ocean from the whole Mississippi river basin) or individual small watersheds for local applications. There is a lack of spatially explicit data synthesis that covers a large area and resolves spatial heterogeneity at fine scales. New data of this kind are particularly useful because the anthropogenic sources and drivers controlling nitrogen loads often vary across different river reaches or basins^12^; they are instrumental in quantifying nitrogen accrual or depletion at distinct river sections and thereby, advancing our understanding of the underlying mechanisms and contributing factors. However, such data is not readily available yet to the scientific and policy communities, even for data-rich regions such as the United States. In particular, derivation of such data needs to account for river network and hydrological connectivity across the region. A comprehensive delineation of the spatial distribution of nitrogen yields from upstream land areas remains notably absent.

In addition, existing databases focused mostly on riverine nitrogen loads without accounting for the nature of the sources or only on one specific anthropogenic driver such as fertilization or urban development^8,13^. The existing database also rarely differentiated point from non-point sources. The differentiation is important for tracking nitrogen fates across managed landscape and is expected to be instrumental in formulating more efficacious policies to manage soil nutrients and ameliorate water quality. As such, there is also an urgent need of nitrogen pollution database to assess the critical source area of nitrogen loads from non-point (i.e., diffuse pollution from different land uses and atmospheric deposition) and point sources (e.g. concentrated discharge from sewage treatment plant or chemical plant).

**Fig. 1:**
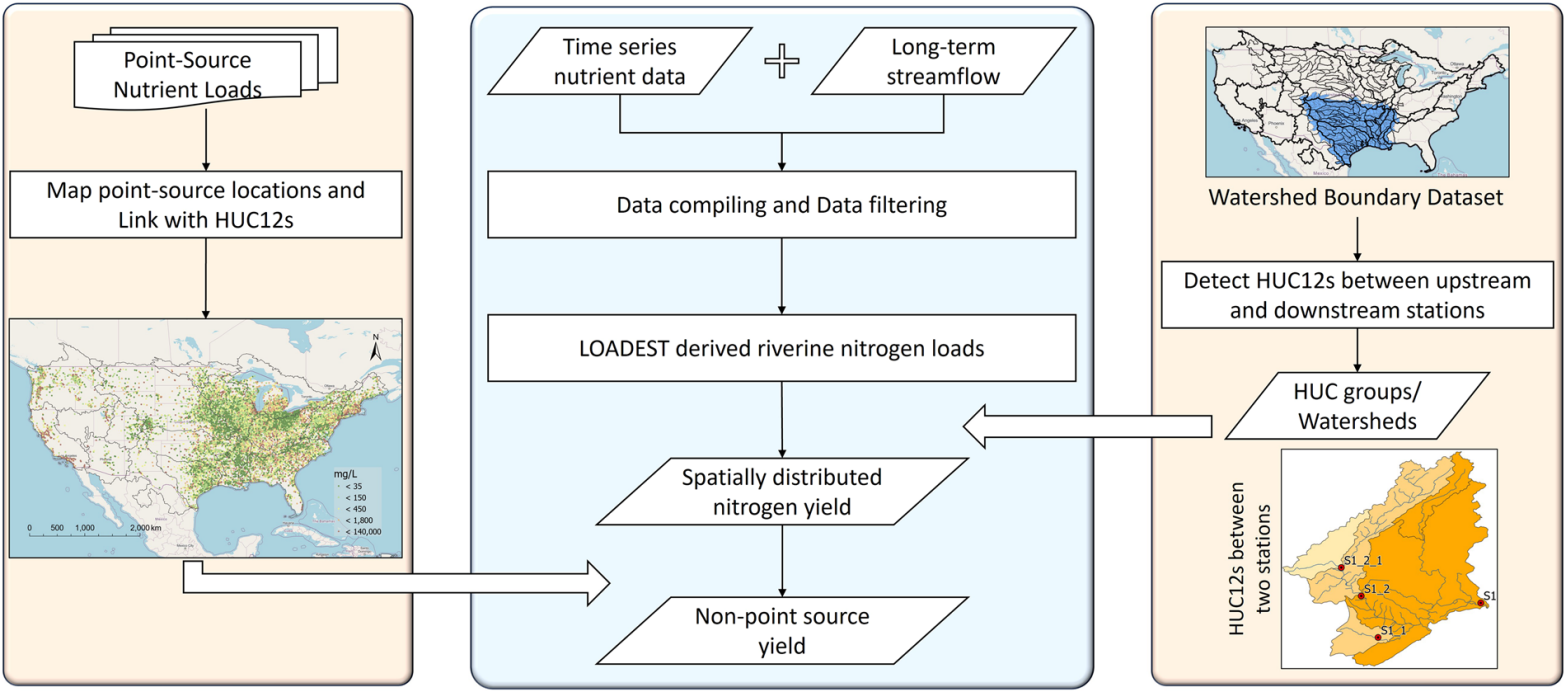
Overview of the generation of nitrogen load data across the CONUS.

We aim to address the gaps in the available nitrogen pollution databases by deriving riverine nitrogen loads (Fig. 1) at more than 1,800 hydrological monitoring stations distributed across the Conterminous United States (CONUS). By leveraging the upstream-downstream river connectivity for catchments from the NHDPlus Hydrologic Unit Code 12-digit (HUC12) geospatial dataset, we computed the riverine nitrogen yield, which is the net gain or loss of riverine nitrogen between upstream and downstream hydrological stations divided by the controlled watershed area. To further elucidate non-point source contributions to the riverine nitrogen loads, we removed the point source contributions in the recently published “Point-Source Nutrient Loads to Streams of the Conterminous United States” dataset^14^. This new dataset facilitates a more spatially resolved analysis of critical source areas of nitrogen pollution in the CONUS. Such dataset also helps understand control factors of riverine nitrogen gain or loss across different social and physiographic regions, as well as provides observational data for verifying credibility of watershed water quality models that are often used to support nitrogen pollution mitigation.

## Methods

### Data acquisition and preprocessing

To estimate nitrogen loads, we compiled collocated and concurrent daily records of both streamflow and nitrogen concentrations from 1954 to 2023 in 1,861 hydrologic stations across the CONUS. The streamflow data were sourced from the United States Geological Survey (USGS) National Water Information System (NWIS). We also downloaded nitrogen concentration data from the Water Quality Portal (https://www.waterqualitydata.us/), which is a hub of water quality data from an array of authoritative sources including the USGS, Environmental Protection Agency (EPA), as well as more than 400 state, federal, tribal, and local agencies. Note that we only used the nitrogen concentration data from USGS that are collocated and concurrent with observed streamflow data from the same USGS station. For nitrogen concentration data, when multiple records were available on a single day, we computed the average rather than a flow-weighted average due to the use of daily streamflow data. Moreover, if multiple stations available in one HUC12, we only retained the station closest to the basin outlet in the mainstem.

### Estimation of Nitrogen Loads Using LOADEST

**Fig. 2:**
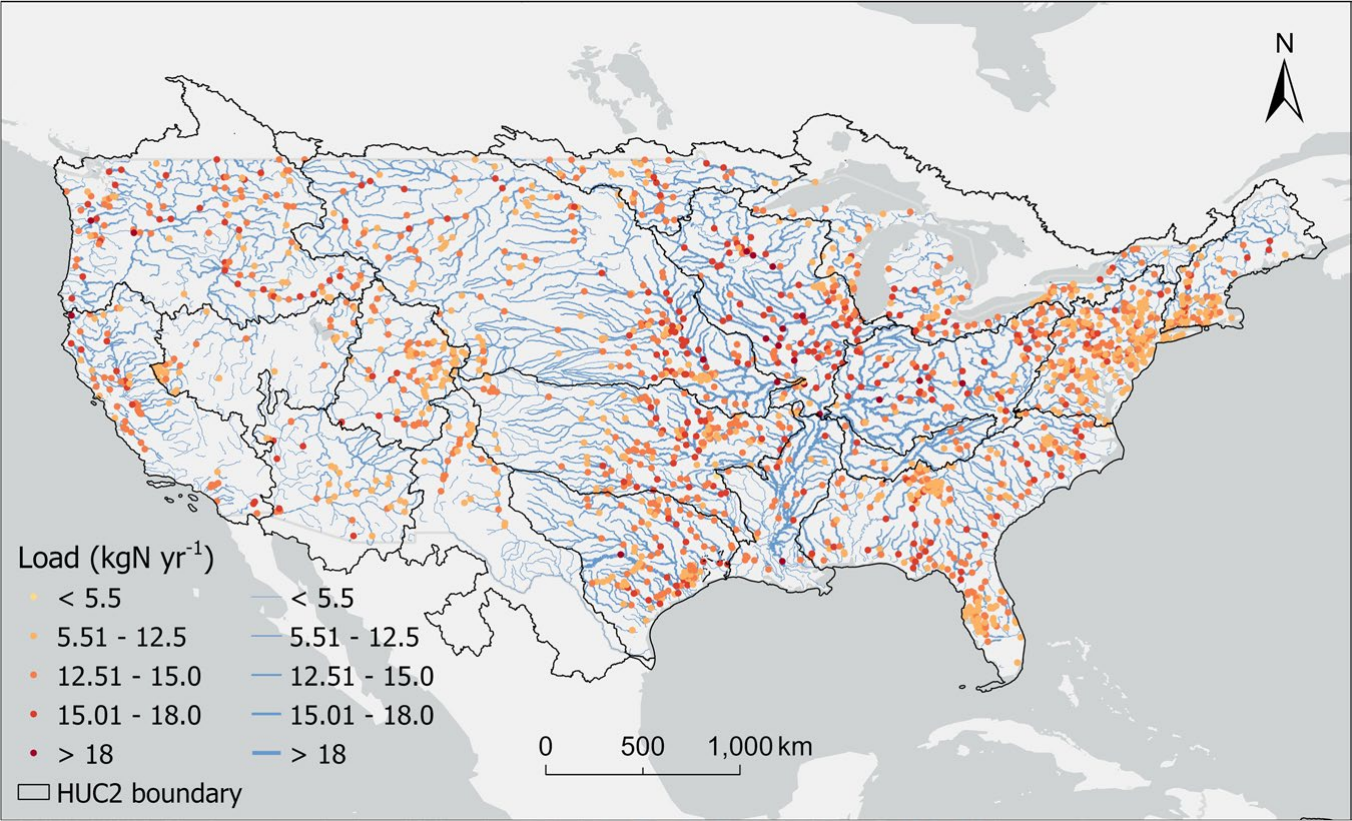
Riverine TN loads across the CONUS calculated by the LOADEST program. Points represent the average TN loads at USGS stations, which are further linearly interpolated along the mainstem of the flowlines (i.e., the longest flow path in each HUC8) to show the changes in TN loads along river networks. Due to the skewness of the data toward high values, we applied a logarithmic transformation ($\mathrm{ln}\left(1+x\right)$,where $x$ is the TN load) for visualization purpose.

We employed LOADEST, a FORTRAN-based computer program designed for the estimation of constituent loads in streams and rivers using the Adjusted Maximum Likelihood Estimation (AMLE) approach, to compute the nitrogen loads at individual hydrological stations (Fig. 2)^15^. We excluded stations with fewer than 12 paired streamflow-nitrogen concentration data records to meet the statistical analysis requirements of LOADEST. Nine predefined regression models (Table 1) were compared to identify the most reliable equation for each individual hydrologic station. The selection of the best model was based on the Akaike Information Criteria (AIC), which integrates both maximum likelihood and the count of independently adjusted parameters in the model^16^. In LOADEST, “load” refers to the total mass of nitrogen over a specified time period. The initial output from LOADEST is measured in grams per day, which we convert to kilograms per year. We calculated different nitrogen forms including total nitrogen (TN), total organic nitrogen (TON), ammonium (NH_4_^+^) and nitrate (NO_3_^−^). For example, 294,996 records of daily TN concentration from 1,861 stations were used. The average TN load (1,916,464 kgN yr^−1^) is much higher than the median value (260,532 kgN yr^−1^), indicating that a subset of hydrological stations exhibits exceptionally high values. The exact temporal spans and the total counts of available observations at each station are described in the dataset provided.

**Table 1:** Regression models in LOADEST.

ID	Regression model
1	${a}_{0}+{a}_{1}\,\mathrm{ln}\,Q$
2	${a}_{0}+{a}_{1}\,\mathrm{ln}\,Q+{a}_{2}\,\mathrm{ln}\,{Q}^{2}$
3	${a}_{0}+{a}_{1}\,\mathrm{ln}\,Q+{a}_{2}{dtime}$
4	${a}_{0}+{a}_{1}\,\mathrm{ln}\,Q+{a}_{2}\,\sin (2\pi {dtime})+{a}_{3}\,\cos (2\pi {dtime})$
5	${a}_{0}+{a}_{1}\,\mathrm{ln}\,Q+{a}_{2}\,\mathrm{ln}\,{Q}^{2}+{a}_{3}{dtime}$
6	${a}_{0}+{a}_{1}\,\mathrm{ln}\,Q+{a}_{2}\,\mathrm{ln}\,{Q}^{2}+{a}_{3}\,\sin \left(2\pi {dtime}\right)+{a}_{4}\,\cos \left(2\pi {dtime}\right)$
7	${a}_{0}+{a}_{1}\,\mathrm{ln}\,Q+{a}_{2}\,\sin \left(2\pi {dtime}\right)+{a}_{3}\,\cos \left(2\pi {dtime}\right)+{a}_{4}{dtime}$
8	${a}_{0}+{a}_{1}\,\mathrm{ln}\,Q+{a}_{2}\,\mathrm{ln}\,{Q}^{2}+{a}_{3}\,\sin \left(2\pi {dtime}\right)+{a}_{4}\,\cos \left(2\pi {dtime}\right)+{a}_{5}{dtime}$
9	${a}_{0}+{a}_{1}\,\mathrm{ln}\,Q+{a}_{2}\,\mathrm{ln}\,{Q}^{2}+{a}_{3}\,\sin \left(2\pi {dtime}\right)+{a}_{4}\,\cos \left(2\pi {dtime}\right)+{a}_{5}{dtime}+{a}_{6}{{dtime}}^{2}$

Note: ln *Q* is the difference between ln *streamflow* and *center of* ln (*streamflow*); *dtime* is the difference between *decimal time* and *center of decimal time*; *a*_0_, *a*_2_, *a*_3_, *a*_4_, *a*_5_, and *a*_6_ are model regression coefficients.

### Watershed yield of riverine nitrogen

After calculating the nitrogen loads at each individual hydrological stations, we further leveraged the upstream-downstream topology information contained in the Hydrologic Unit Catalogue – 12 (HUC12) catchments of the National Hydrography Dataset Plus (NHDPlus) dataset (https://nhdplus.com/NHDPlus/NHDPlusV2_data.php) to estimate the net gain or loss nitrogen in watersheds between different pairs of upstream-downstream hydrological stations.

We derived spatial riverine nitrogen yield for watersheds between upstream-downstream hydrological stations using the approach described in Qiu *et al*.^17^. This process commenced with the identification of the specific HUC12 within which each hydrological station is situated. The most downstream HUC12 encompassing the hydrological station was designated as S1 (the first-level or the most downstream station). Subsequently, the tracing of upstream HUC12s was conducted until reaching one or multiple HUC12 catchments housing a hydrological station. These upstream HUC12 catchments and hydrological stations were assigned second level labels, such as S1_1, S1_2, and S1_3. The HUC12s falling within the ambit of the first-level station (S1) and the second-level stations (S1_1, S1_2, S1_3, and so on) were grouped as one watershed and marked as a unique “S1” HUC group. The net gain or loss of riverine nitrogen yield from the “S1” watershed was computed by dividing the discrepancy between the load at the first-level station and the aggregated load from the second-level stations by the area of the “S1” watershed. Next, we designated each hydrologic station from the second-level stations as a first-level station and repeat the same procedures described above. Those procedures are iterated until all hydrological stations are processed, resulting in the spatial nitrogen yield maps over the CONUS.

**Fig. 3:**
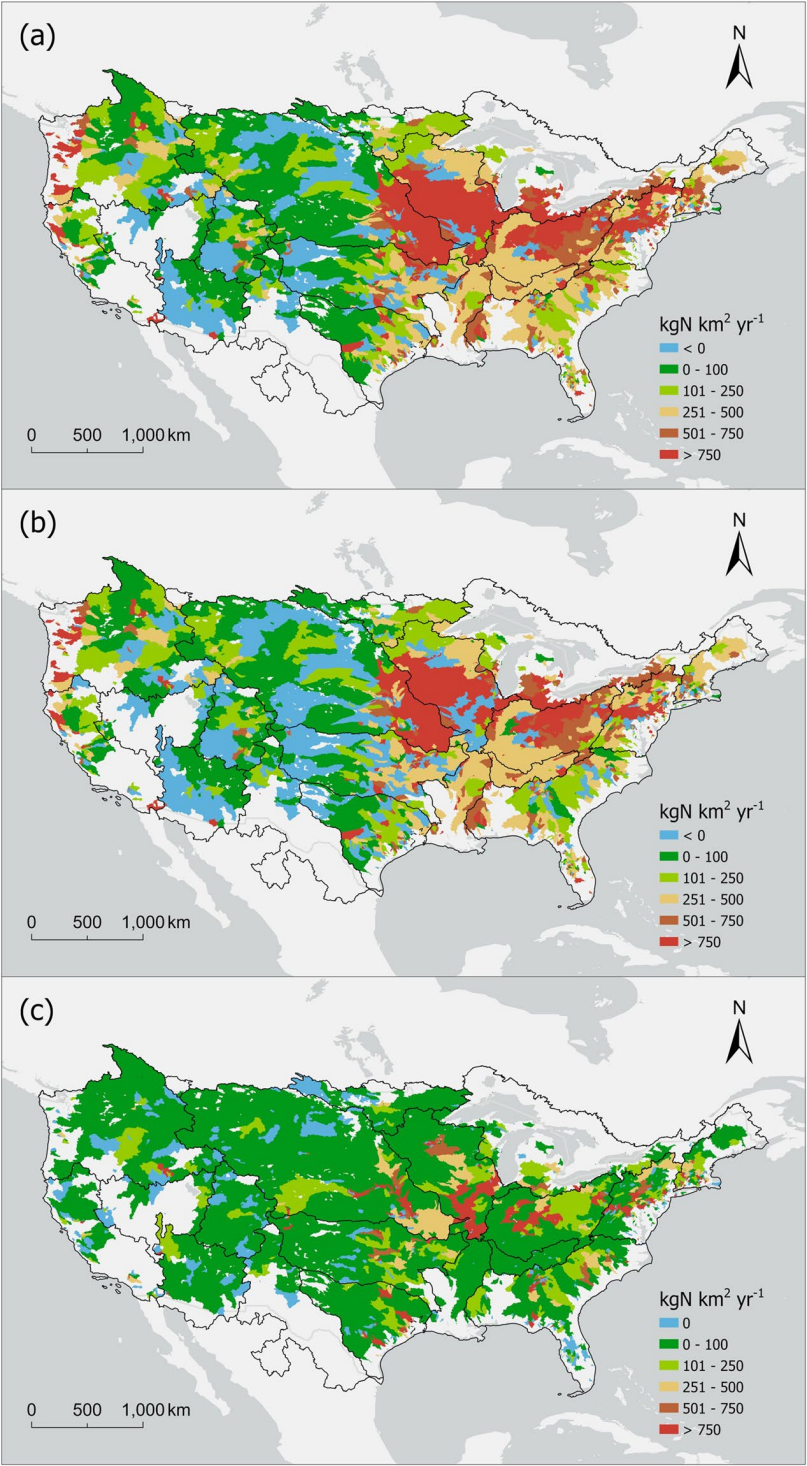
Spatial distribution of watershed (**a**) TN yield, (**b**) non-point source TN yield, and (**c**) point source TN yield across the CONUS.

At the HUC12 level, there are a total of 86,744 catchments located within the CONUS. As some HUC12 catchments do not drain to any hydrological stations, the resultant long-term average watershed TN yield map (Fig. 3a) encompasses 66,694 HUC12 catchments. The map shows that U.S. Midwest and Northeast have the highest TN yield and are the major sources of nitrogen pollution. The spatial patterns of other nitrogen forms are similar to the TN yield pattern (Fig. S1).

### Nonpoint-source contributions to riverine nitrogen yield

The watershed nitrogen yield maps help illustrate the gain or loss of riverine nitrogen from both non-point and point sources. Nonpoint-source nutrient loads, primarily originating from surface runoff and precipitation and notably influenced by agricultural practices and energy production^18,19^, often constitute the predominant cause of water quality issues. To discern nonpoint-source contributions, we removed the point source contributions from the “Point-Source Nutrient Loads to Streams of the Conterminous United States” dataset^14^. This dataset encompasses 5,430 major point-source facilities, such as wastewater treatment plants, chemical plants, and landfills, alongside 11,537 minor wastewater treatment facilities discharging into streams within the CONUS during the year 2012. In total, 16,967 point-source facilities are distributed across 11,601 HUC12 catchments, primarily concentrated in the eastern CONUS. We estimated point-source TN load for each HUC12 catchment by aggregating loads from all point-source facilities within that HUC12.

After removing the point-source contributions (Fig. 3c), we estimated non-point source TN yield (Fig. 3b). In general, point-source contributions are relatively small compared to that of non-point sources but can noticeably change the spatial distribution of watershed TN yield. Large point-source TN yields are primarily found in the Midwest and Southeast regions, which typically originate from wastewater treatment plants and petroleum refineries, two major sources of point discharge^14^. By removing point-source contributions, the area of watersheds with negative yield or loss of riverine TN increased from 1,043,641 to 1,432,602 km^2^. Over the CONUS, the area-weighted TN yield is 532.5 and 341.02 kgN km^−2^ yr^−1^, respectively, with and without point-source contributions. This indicates that point sources contributed ca. 36% of the riverine TN yield. However, this is likely the upper bound estimate of the point source contributions as part of the point source inputs will be removed through riverine processes^20^.

## Data Records

The “Spatial Nitrogen Yield” Dataset is accessible through Wang *et al.*^21^. It encompasses three components: (1) Nitrogen Loads at Hydrological Stations, (2) Watershed Nitrogen Yield, and (3) Nonpoint-Source Nitrogen Yield.

The “Nitrogen Loads” dataset contains the TN loads at each hydrological station (Table 2). To better illustrate estimation uncertainty, we also provide the lower and upper nitrogen load values for the 95% confidence intervals, which were calculated using the standard error of prediction. Furthermore, this dataset includes additional information, such as the start and end years of the observational data, as well as AIC and coefficient of determination (R-Squared) for the selected LOADEST model. These attributes offer users flexibility in sub-setting the dataset based on their specific research requirements.

**Table 2:** Data records contained in the “Nitrogen Loads” datasets.

Field name	Definition
Station ID	U.S. Geological Survey designated ID
Lat	Latitude of the current station
Long	Longitude of the current station
TN load	The amount of average nitrogen loads from the hydrologic station (kgN yr^−1^)
lowerB	Lower bound of the 95% confidence interval (kgN yr^−1^)
upperB	Upper bound of the 95% confidence interval (kgN yr^−1^)
obsNum	The number of paired nitrogen concentration with streamflow data
cntrTime	Center of decimal time
startYr	The starting year of the observed data
endYr	The ending year of the observed data
modelID	The ID of regression modeled used for load estimation
AIC	AIC value
r2	R-Squared (%)

The “Watershed Nitrogen Yield” dataset comprises nitrogen yield at the HUC group scale (Table 3). The first sheet in the dataset contains average watershed yield for various nitrogen forms, and other sheets includes the annual nitrogen yield from 1981 to 2022. However, due to limited availability of concentration data for a specific year, the spatial coverage of annual nitrogen yield data is not as extensive as long-term average watershed yield data (Fig. S2). This dataset lends itself to enhanced integration with other spatial datasets, such as the NHDPlus and the WBD, effectively contributing to the enrichment of the preexisting national hydrography repositories. Broadly, we observe that within the CONUS, there are 55,671 HUC12s featuring TN yield greater than zero. These HUC12s exhibit an increasing spatial pattern from west to east, while TN loads are also high in the western coastal areas. Eastern areas characterized by heightened TN yields are concentrated in the Midwest and eastern regions, notably prevalent in the upper Mississippi basin and Ohio area, where intensive agricultural activities predominate. Conversely, regions situated in the western CONUS reflect lower nitrogen yields, although elevated nitrogen yields are identified in proximity to the northern coastal regions. The “Nonpoint-Source Watershed Nitrogen Yield” dataset is distinctive in its exclusion of point-source TN.

**Table 3:** Data records contained in the “Watershed Nitrogen Yield” and “Nonpoint-Source Watershed Nitrogen Yield” datasets.

Field name	Definition
HUC12	12-digit ID for each HUC from the WBD
TN	Average watershed yield of total nitrogen (kgN km^−2^ yr^−1^)
TON	Average watershed yield of total organic nitrogen (kgN km^−2^ yr^−1^)
NH4	Average watershed yield of ammonium (kgN km^−2^ yr^−1^)
NO3	Average watershed yield of nitrate (kgN km^−2^ yr^−1^)

Note that for the latter dataset, only HUC12 and TN are available.

## Technical Validation

**Fig. 4:**
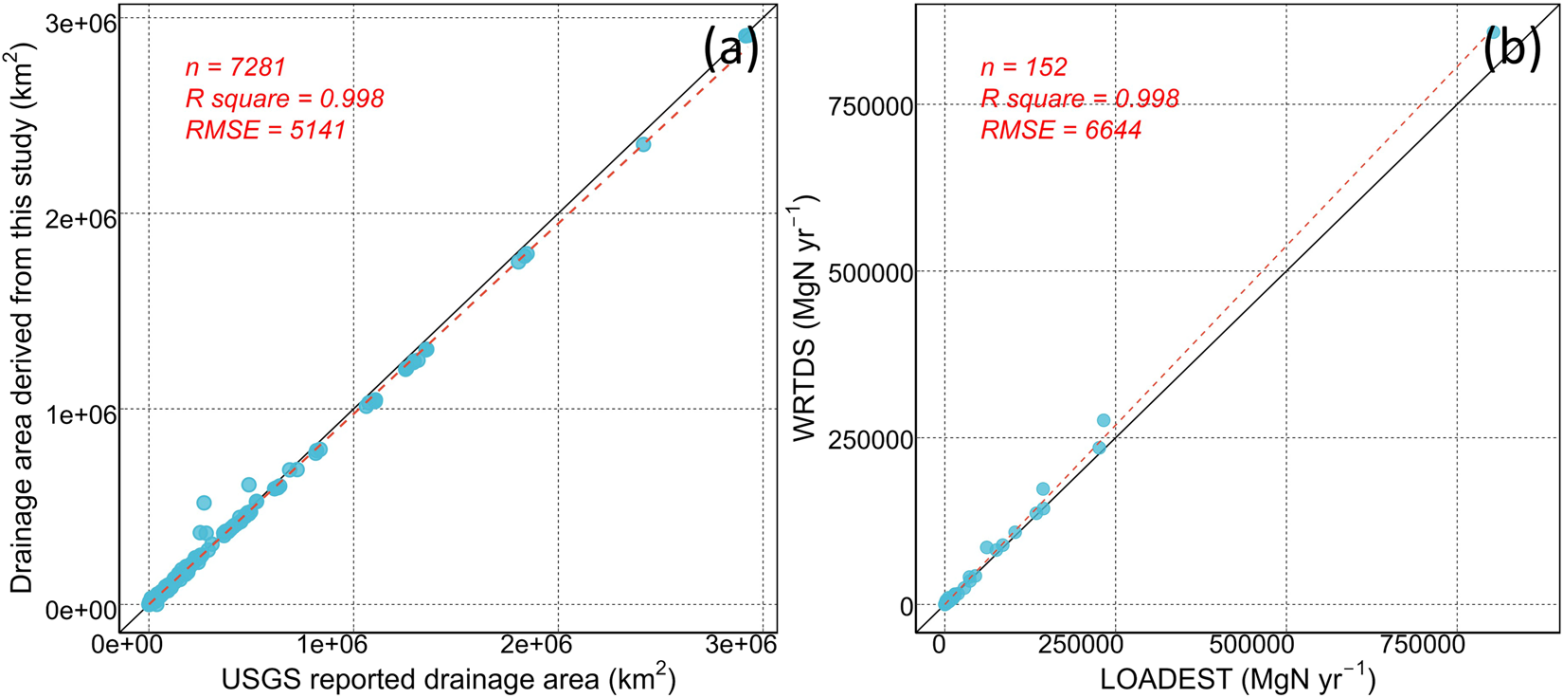
Validation of drainage area and estimated TN loads. (**a**) Comparison between drainage area derived from revised WBD and the USGS GAGES-II dataset; (**b**) Comparison between average monthly TN estimated by LOADEST and TN from WRTDS.

To ascertain the precision of our data, we conducted a comparative analysis between the drainage area (Fig. 4a) and the drainage area data extracted from the USGS GAGES-II dataset^22^. In this comparison, when multiple USGS stations were situated within a single HUC12, we specifically retained the principal stem hydrological stations and omitted tributary stations. Notably, the majority of data points align closely along the 1:1 line. This agreement, coupled with a high R-square value, underscores the substantial concordance between the drainage area derived from the improved WBD and the USGS GAGES-II dataset. This robust agreement serves to affirm the reliability of our rectified upstream-downstream topology information.

To assess the derived riverine nitrogen loads, we compared the LOADEST derived estimates against an independent riverine TN load dataset derived using the Weighted Regressions on Time, Discharge, and Season (WRTDS) model^23^. A total of 152 hydrological stations with both WRTDS and LOADEST derived data were compared (Fig. 4b). The R-square value closely approximates unity, whereas the Root Mean Square Error (RMSE) is under 7,000 MgN yr^−1^. The minor discrepancies noted are likely attributed to variations in the temporal periods covered by the two datasets. This result substantiates the reliability of the riverine TN loads derived from this study.

## Usage Notes

The methods and data records sections provide information for readers to acquire a comprehensive grasp of the methodologies employed for the estimation and arrangement of nitrogen loads. This foundational understanding shall empower users to optimize their utilization of the nitrogen dataset. Furthermore, given disparities in temporal coverage, availability of concentration data records, and the accuracy of regression models, it is imperative for users to judiciously select specific subsets of nitrogen loads that align with their distinct research requisites.

In a broader context, the new nitrogen load and watershed yield datasets can serve as versatile resources across an array of research domains:

1. The “Nitrogen Loads at Hydrological Stations” dataset, structured at the level of hydrological stations, can form the basis for large-scale watershed model evaluations^24^.
2. The “Watershed Nitrogen Yield” dataset, when conjoined with supplementary datasets encompassing climate, land cover, and soil properties, can be utilized to scrutinize the dominant factors influencing the net gain or loss of various nitrogen constituents. Further insights into environmental controls on riverine nitrogen loads can be garnered by considering hydraulic attributes, including reach length, width, depth, skewness, as well as streamflow data sourced from the NHDPlus and other relevant repositories.
3. The “Watershed Nitrogen Yield” dataset, with its capacity to depict the spatial distribution of nitrogen yield, aids researchers in constructing comprehensive riverine nitrogen budgets. This spatial nitrogen yield data, in turn, facilitates estimations of nitrogen fluxes traversing from rivers to the ocean across the CONUS. Comparative analyses of upstream-downstream topological relationships enable assessments of the nitrogen removal potential, thereby providing valuable insights for policymakers in formulating more effective decisions.
4. Nonpoint sources, predominantly influenced by human activities, exert significant control over riverine nitrogen fluxes toward coastal regions^25^. The “Nonpoint-Source Nitrogen Yield” dataset equips policymakers with the tools to discern regions where nitrogen loads are most substantially influenced by commercial fertilizer application and animal manure^26^.

The dataset derived in this study encapsulates historical average conditions grounded in observations at numerous hydrological stations. Regions characterized by sparse distribution of hydrological stations, particularly evident in southwestern areas, are devoid of nitrogen load coverage. Future research endeavors may explore the integration of machine learning techniques and the fusion of process-based and statistical models to enhance coverage and the explanatory depth in ungauged basins.

### Supplementary information

SI A dataset of riverine nitrogen yield across watersheds in the Conterminous United States

## Data Availability

All the codes for processing the nitrogen yields were calculated using R version 4.3.1 and archived at GitHub: https://github.com/ymwang4924/tn.git.
