# Supplementary material for: A dataset of riverine nitrogen yield across watersheds in the Conterminous United States
Source: Sci Data. 2024 Jun 29;11:712. doi: 10.1038/s41597-024-03552-1 (PMC11217459; doi:10.1038/s41597-024-03552-1)
Supplement: Supplementary file 1 — SI A dataset of riverine nitrogen yield across watersheds in the Conterminous United States [file 41597_2024_3552_MOESM1_ESM.docx]

**Supplementary Information**

Table of Contents

Fig. S1. Average watershed yield of various nitrogen forms

Fig. S2. An example of the spatial coverage of annual nitrogen yield


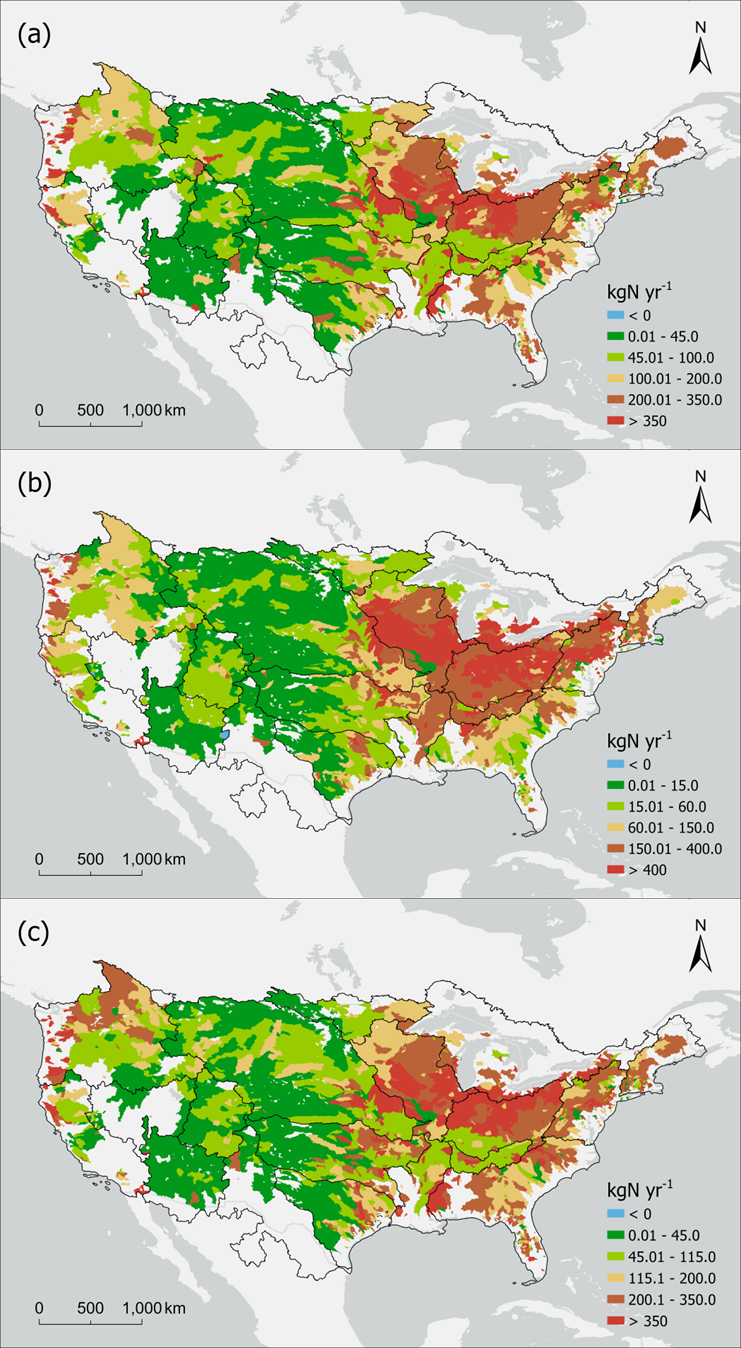


Fig. S1. Average watershed yield of (a) TON, (b) NO_3_^-^, and (c) NH_4_^+^.


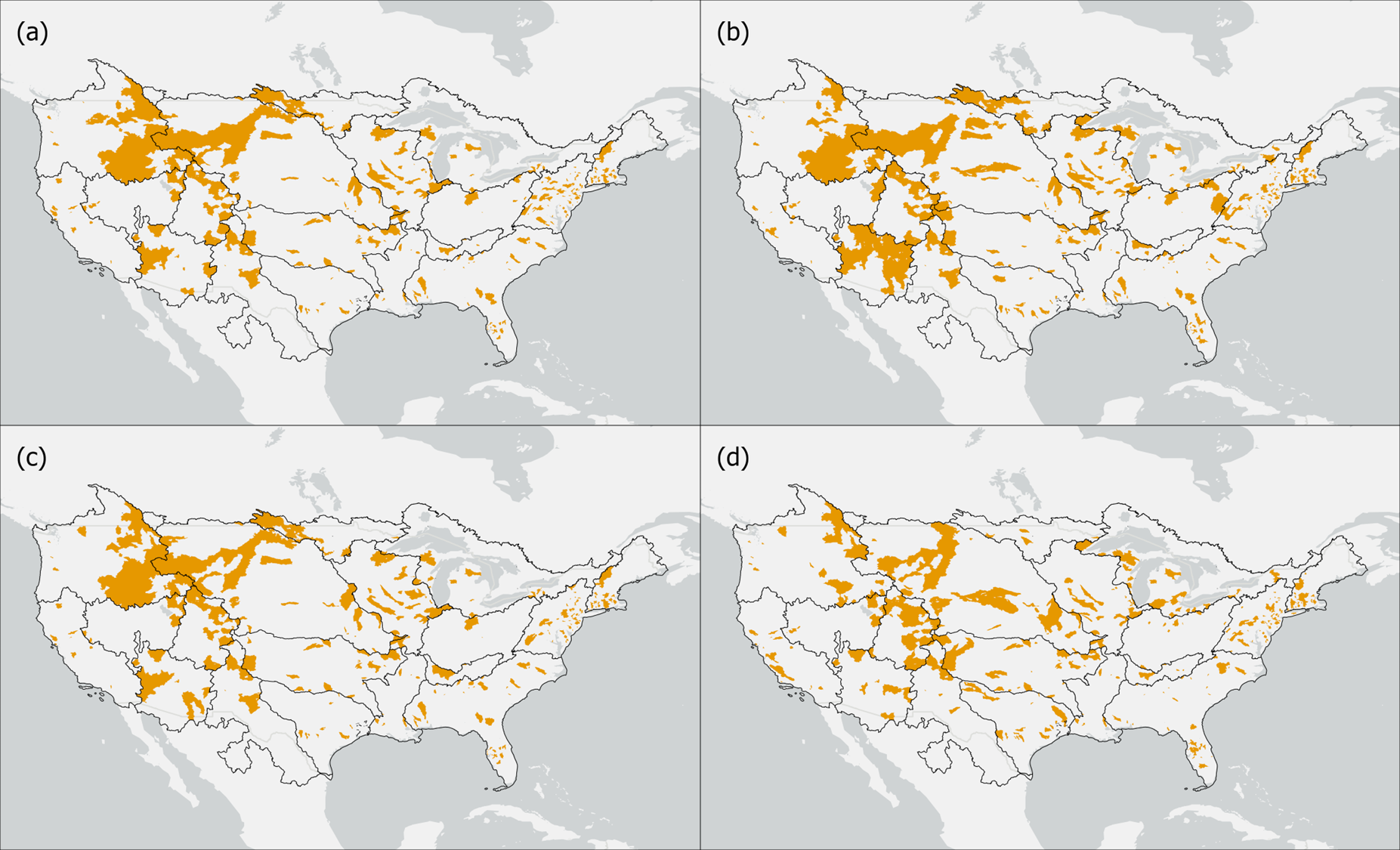


Fig. S2. An example of the spatial coverage of annual nitrogen yield data in 2000 for (a) TN, (b) TON, (c) NH_4_^+^, (d) NO_3_^-^.
